# Supplementary figures and images for: Rates of morphological evolution in Captorhinidae: an adaptive radiation of Permian herbivores
Source: PeerJ. 2017 Apr 13;5:e3200. doi: 10.7717/peerj.3200 (PMC5392250; doi:10.7717/peerj.3200)

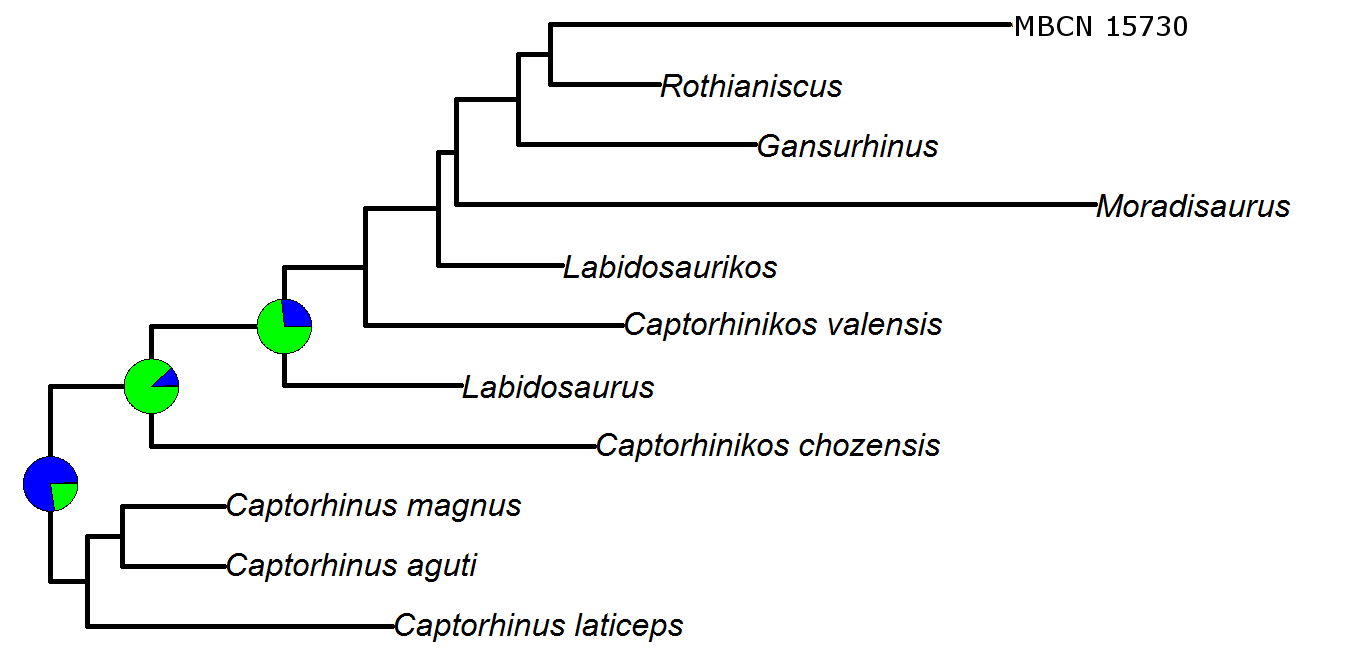

Supplement: Figure S1 — Probabilities of ancestral diets calculated for each of the three nodes for which likelihood gave uncertain results, using Reversible Jump Markov Chain Monte Carlo method. Pie charts on the nodes represent the probabilities of each character state for that node. Green=herbivore; Blue=omnivore; Red=carnivore [file peerj-05-3200-s006.png]

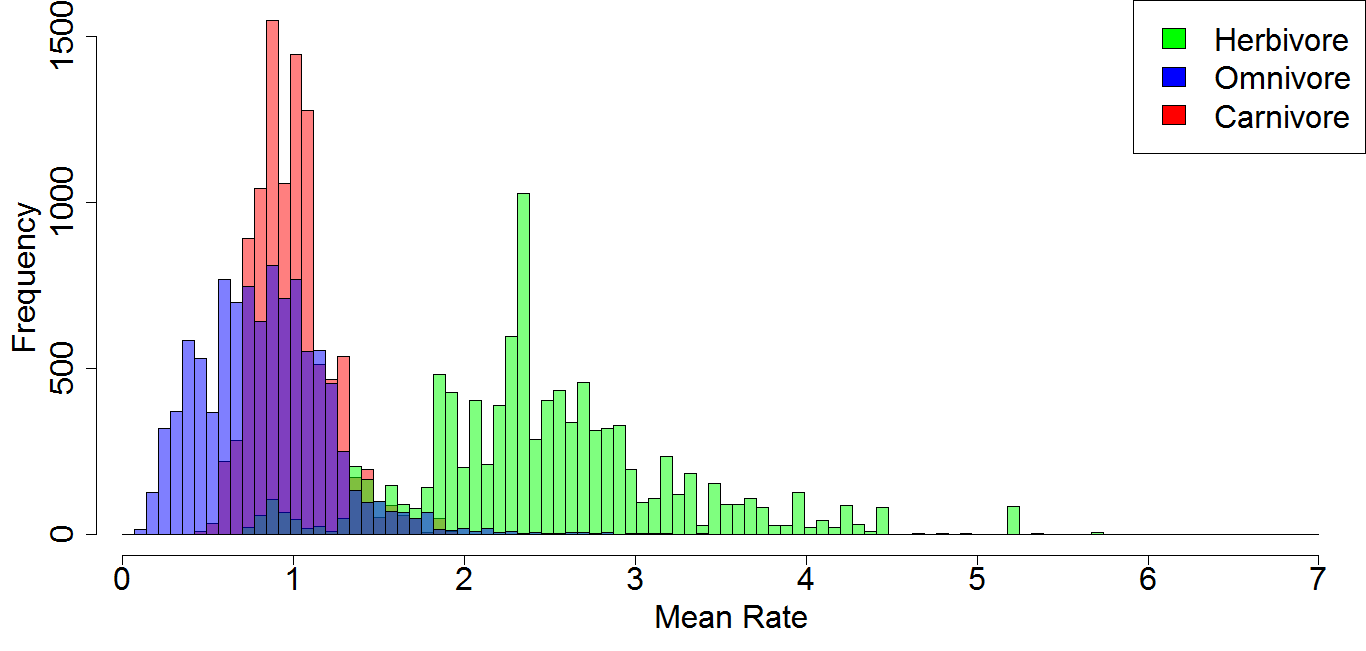

Supplement: Figure S2 — Histogram illustrating the mean rate of discrete character evolution calculate for each dietary regime in each of the 10,000 stochastic maps of dietary evolution. Prior to analysis of rate heterogeneity, three taxa were selected at random to be dropped, in order to test the robusticity of the analyses to sampling variation. [file peerj-05-3200-s007.png]
